# Evolution of metamemory based on self-reference to own memory in artificial neural network with neuromodulation

**DOI:** 10.1038/s41598-022-10173-4

**Published:** 2022-04-26

**Authors:** Yusuke Yamato, Reiji Suzuki, Takaya Arita

**Affiliations:** Graduate School of Informatics, Nagoya University, Nagoya, Japan

**Keywords:** Cognitive neuroscience, Computational neuroscience

## Abstract

The ability of humans to self-monitor and control their memory processes is called metamemory and has been widely studied as a component of metacognition in cognitive psychology. Metamemory in non-human animals has also been investigated in recent years, although it had been regarded as a truly unique characteristic of human memory. We attempt to evolve artificial neural networks with neuromodulation, which have a metamemory function. Our constructive approach is expected to contribute, by introducing a novel dimension of evolutionary plausibility, to the discussion of animal experiments to detect metamemory. In this study, we demonstrate the evolution of neural networks that have a metamemory function based on the self-reference of memory, including the analysis of the evolved mechanism of metamemory. In addition, we discuss the similarity between the structure of the evolved neural network and the metamemory model defined by Nelson and Narens.

## Introduction

**Figure 1:**
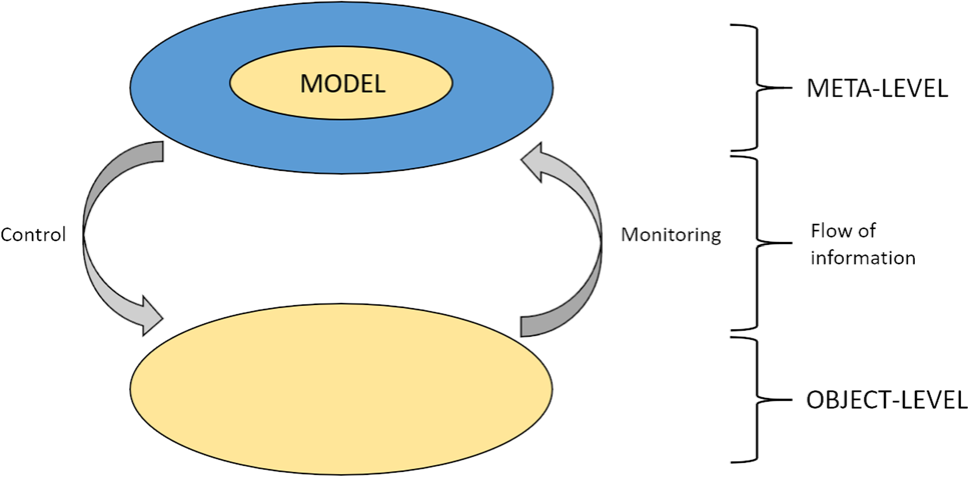
Nelson and Narens’ formulation of a meta-level/object-level theoretical comprising consisting of two structures (meta-level and object-level) and two relationships in terms of the direction of the flow of information between the two levels. The meta-level contains an imperfect model of the object-level.

Metamemory refers to the knowledge and awareness of our own memory processes and has been widely studied as a component of metacognition in cognitive psychology. Based on the formulation by Nelson and Narens, as illustrated in Fig. 1, metamemory is regarded as a complex process in which two cognition layers at different levels of hierarchy are associated by monitoring and control^1,2^. It has been widely studied as a component of metacognition in cognitive psychology^3^. Although it had been regarded as a truly unique characteristic of human memory^4^, testing metacognition or metamemory in animals has become a challenging topic. Furthermore, recent constructive studies that adopt artificial life methods (e.g., evolutionary computation and artificial neural networks) are also being proposed as alternative methodologies. Metacognition is not a behavioral phenomenon but a cognitive process on the living brain. Hence, it is difficult to comprehensively analyze its mechanism in detail. Therefore, comparative studies based on these methodologies, each of which has inherent pros and cons, will become increasingly important in elucidating metacognition.

**Figure 2:**
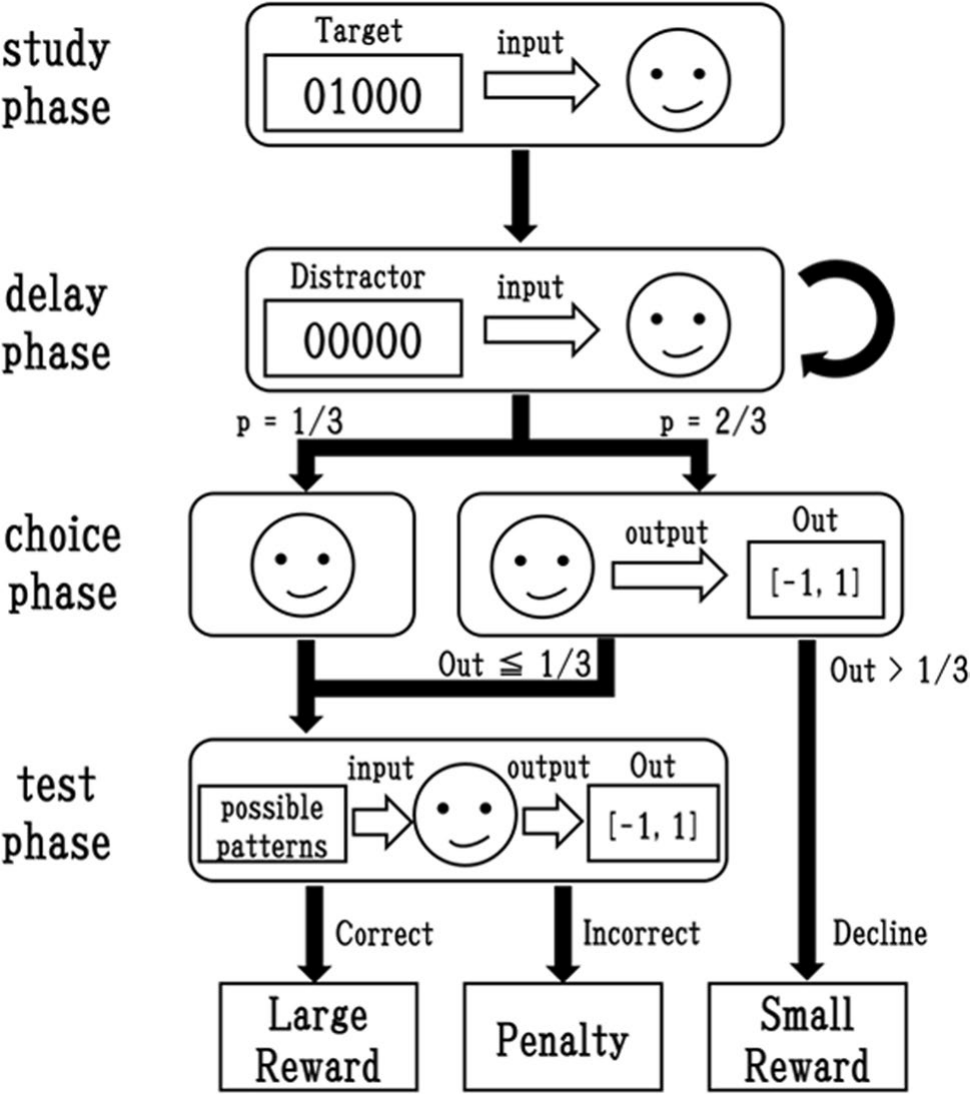
The delayed match-to-sample task that introduced the escape option.

**Figure 3:**
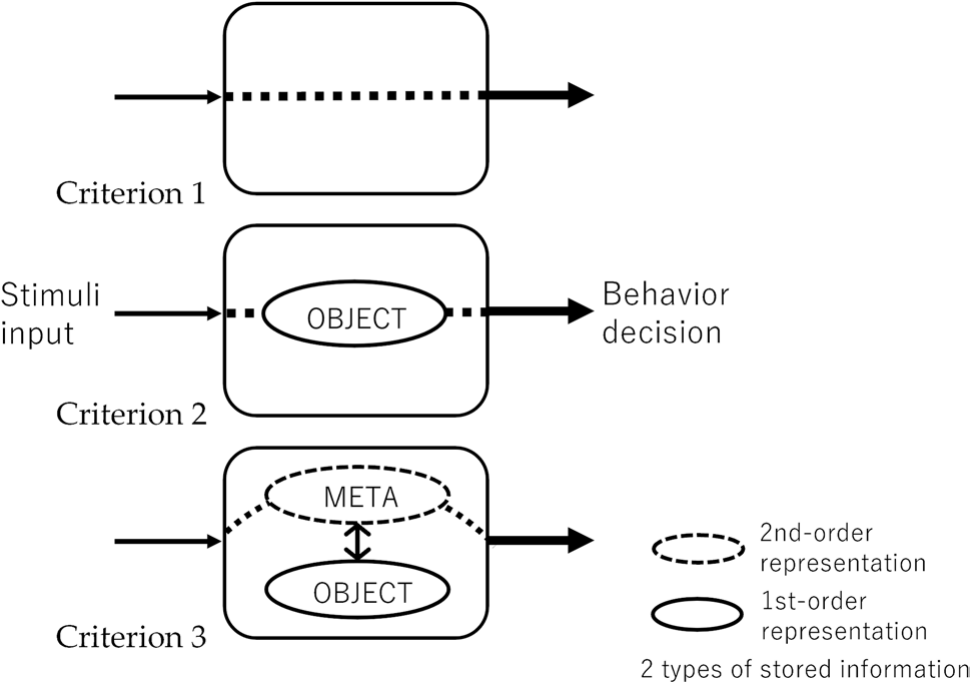
Metamemory criteria.

**Figure 4:**
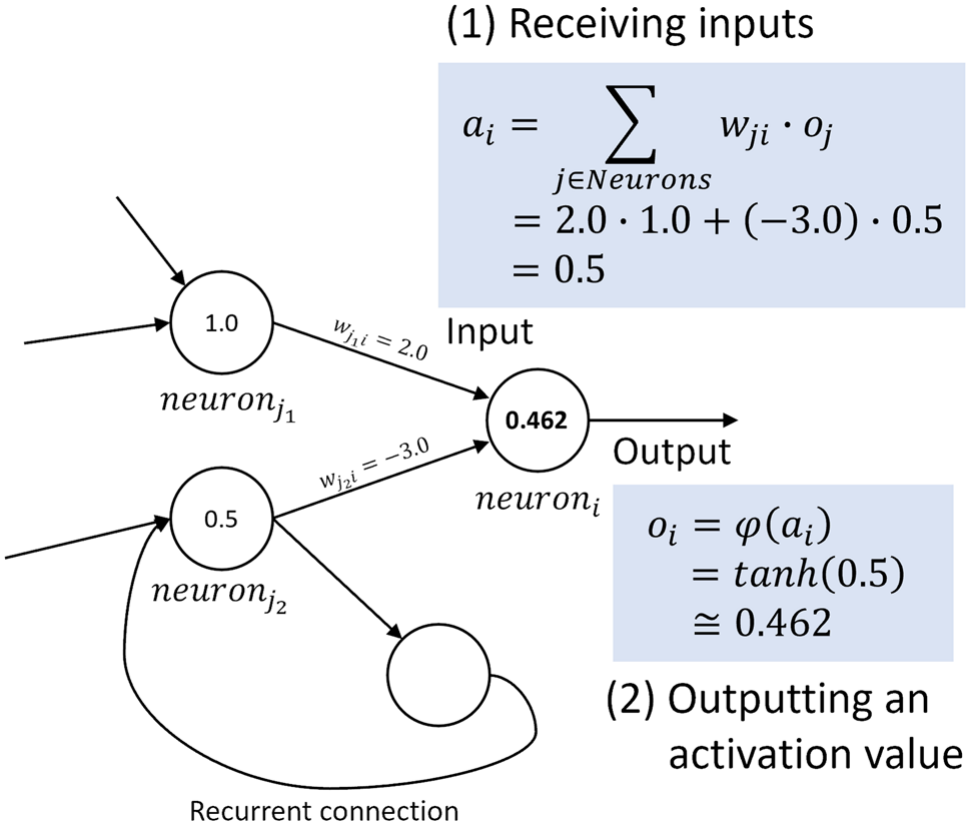
Activation process of a standard neuron in a network.

**Figure 5:**
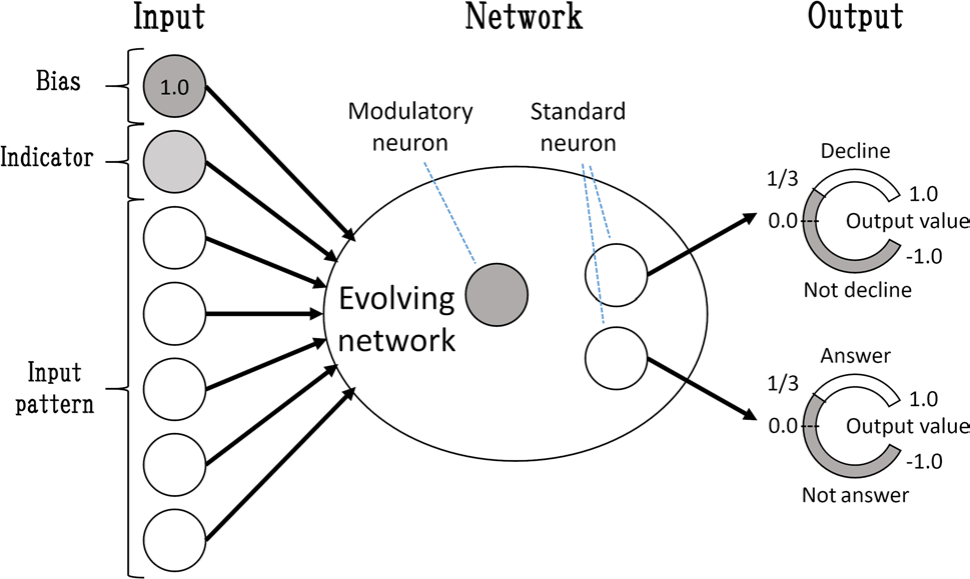
Inputs and outputs of a neural network.

**Figure 6:**
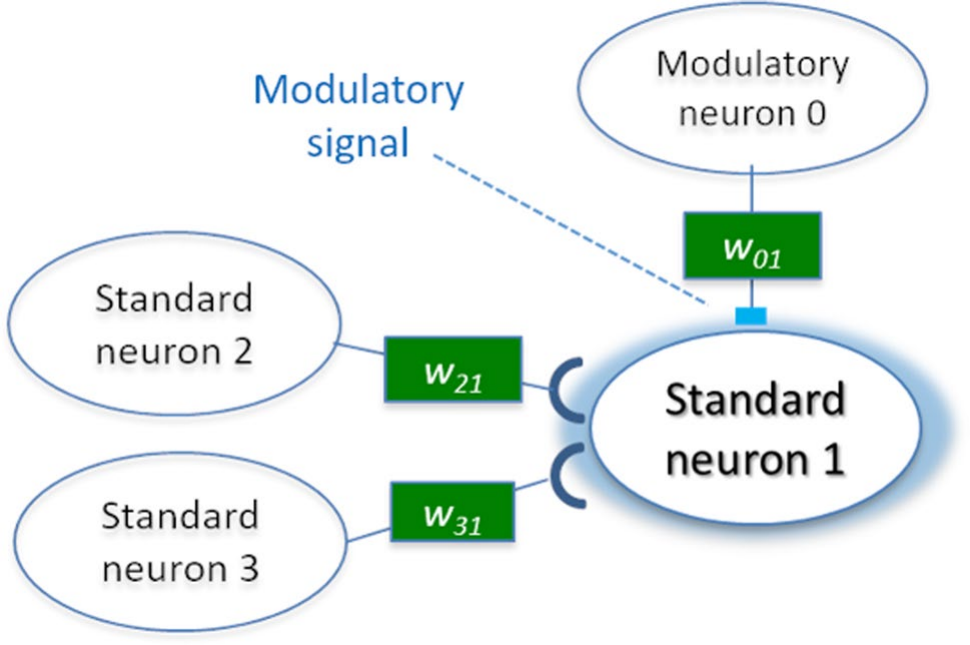
Overview of the neuromodulation.

When we consider animal subjects and investigate whether they have metamemory ability, we have to rely heavily on behavioristic paradigms, as it is impossible to use questionaries as in the case of experiments with human subjects. Hampton devised a *delayed matching-to-sample* (DMTS) paradigm^5^. The idea is as follows: animals that can distinguish between the presence and absence of their own memory should improve accuracy in memory tests if allowed to decline the tests when they have forgotten. They should also decline tests by selecting the escape option more frequently if memory is attenuated experimentally (e.g., by adding forgetting time). Hampton successfully demonstrated that one of the two macaque monkeys examined met these criteria. As a constructive approach, Sudo et al.^6^ used DMTS paradigm and demonstrated that computational agents controlled by artificial neural networks could evolve metamemory ability. Their success partly depended on the usage of a neuromodulation technique^7^ by which a specific type of neurons, *modulatory neurons*, can dynamically alter the plasticity (learning rate) of the connections of the neurons they project to.

However, the validity of the methods in detecting metamemory in animals has sparked widespread discussion and controversy. According to Hampton, it is important that the mechanism of metamemory is categorized into public and private^8^. The former is an adaptive cognitive control that is based on the use of publicly available information, whereas the latter is a cognitive control that is contingent on the privileged access the subject has to their own cognitive states. Hampton pointed out that metamemory detection cannot be inferred to have been achieved unless the availability of publicly accessible external cues is eliminated by focusing on the types of cues used in the metamemory tasks in animal experiments. At the same time, Hampton claimed that his experiments^5^ had detected the metamemory based on internal cues that are private with high probability.

Contrary to Hampton’s claim, other researchers presented interpretations that were independent of internal cues. For example, DMTS-based tests might be passed if the subject selected the escape option when the input pattern seemed difficult to remember, or simply complicated^9^. Furthermore, some researchers have suggested that metamemory tasks like DMTS do not necessarily require metamemory ability, even when the judgment is based on internal cues. For example, taking a strict stance on the definition of the word, metacognition, Carruthers and Crystals distinguished between first-order explanations and metacognition and claimed that alternative explanations based on first-order explanations, instead of metamemory, are possible for the results of many animal experiments for detecting metacognition, including Hampton’s experiment^10,11^. Following this theory, metacognition is essentially cognition about cognition, and should be formed as a second-order representation.

**Figure 7:**
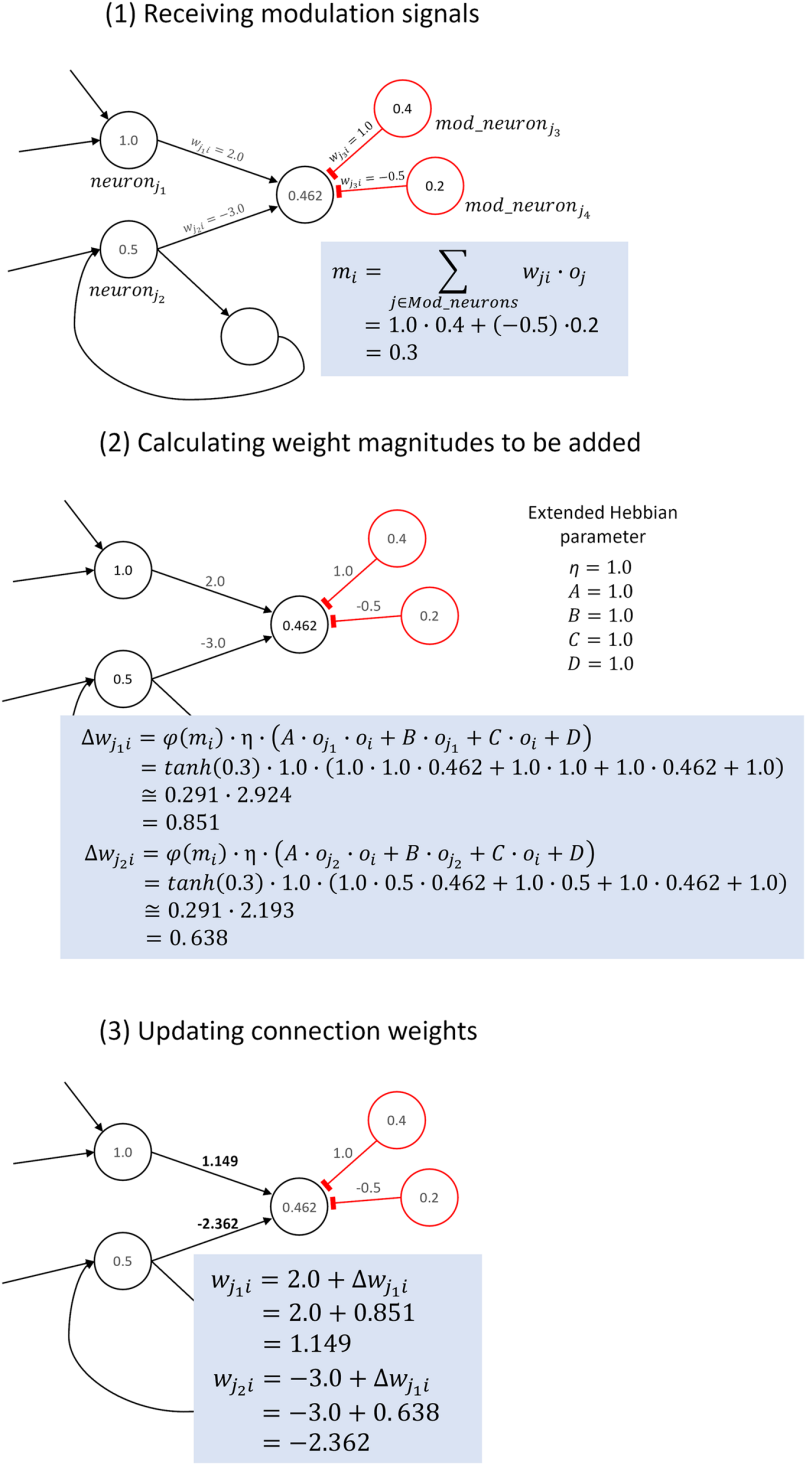
Example of a weight updating process based on both the neuromodulation and extended Hebbian rule.

**Figure 8:**
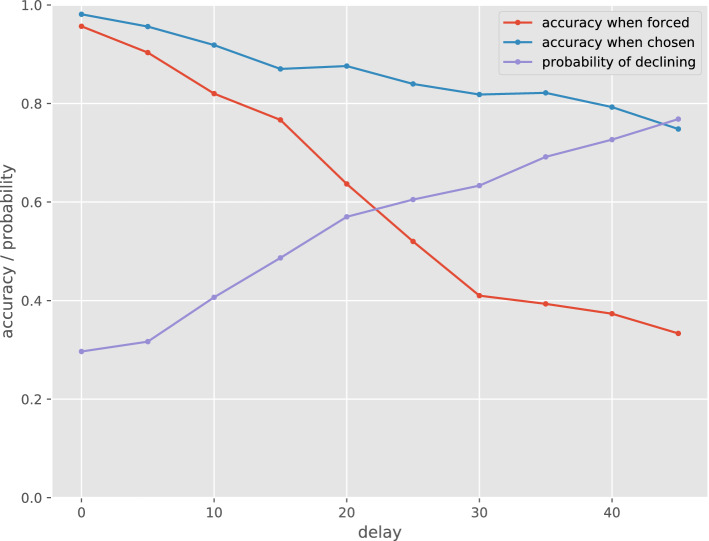
Behavior of the evolved agent for each delay time.

**Figure 9:**
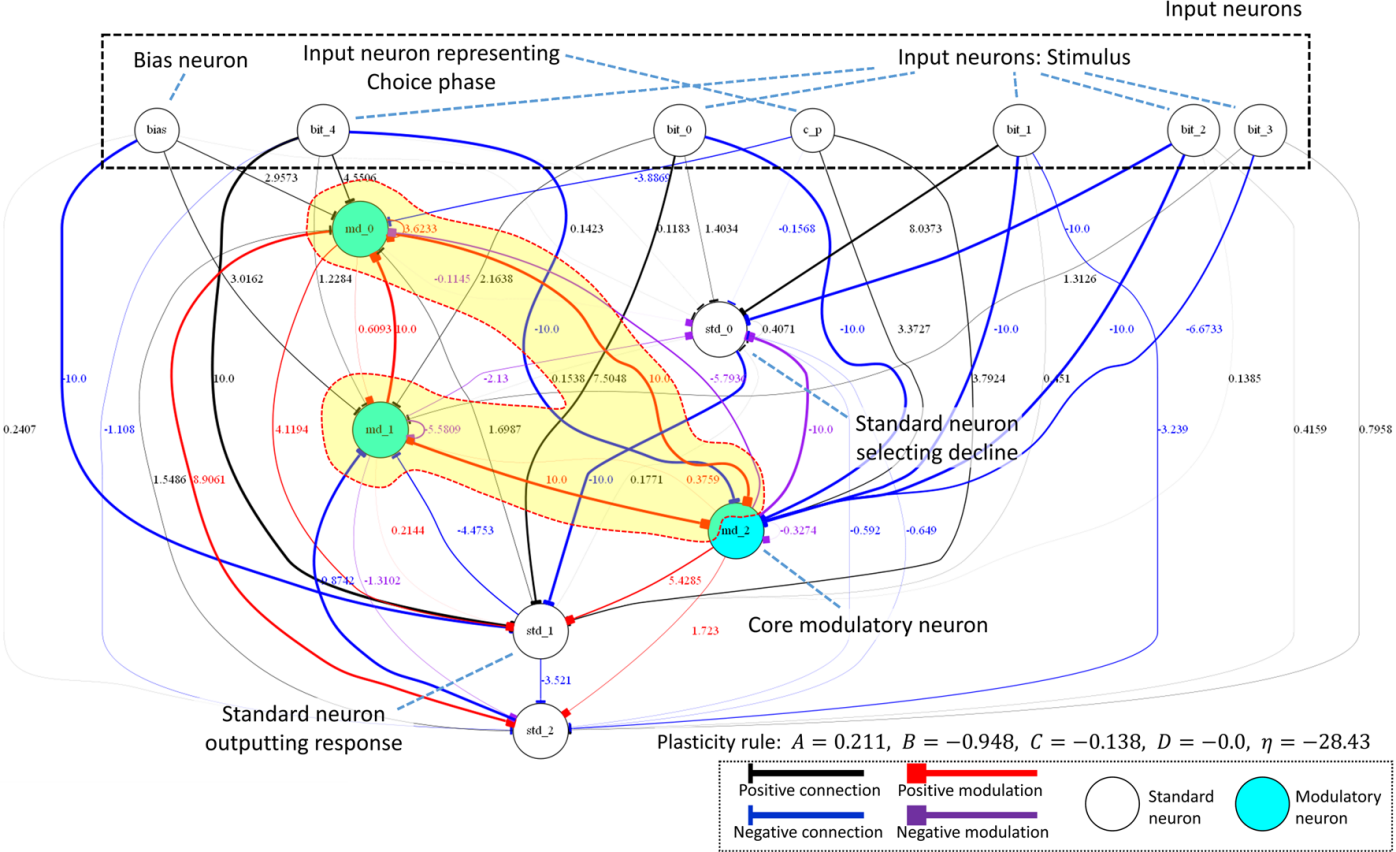
Structure of the evolved neural network. The width of each line represents the weight of a connection.

**Figure 10:**
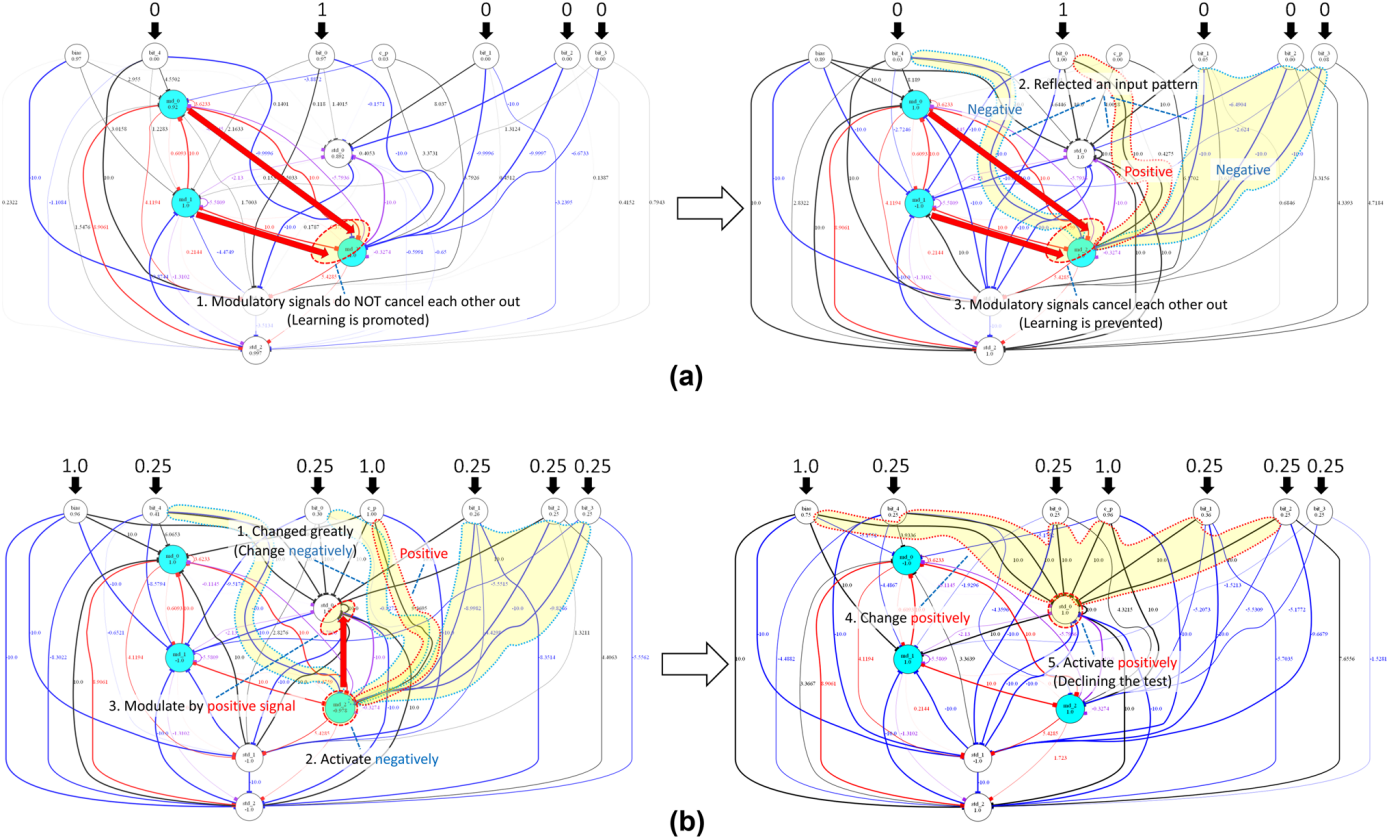
The mechanism by which the network memorizes and maintains a target pattern (00001), and declines the test. Each red arrow represents a modulation. Each event occurs in numerical order. (**a**) The mechanism for memorizing and maintaining a target pattern in the study phase. (1) Learning of connection weights is promoted because the modulatory signal from md_0 to md_2 and that from md_1 to md_2 do not cancel out each other. (2) Consequently, signs of some connections reflect a structure comprising 0 and 1 of an input pattern. (3) Thereafter, the modulatory signals cancel out each other and learning is prevented. In other words, the reflected information is protected. (**b**) The mechanism of declining the test in the choice phase. (1) If the connections in which an input pattern is memorized change significantly, md_2 (core modulatory neuron) receives the inputs in which the sum is negative from bit_0,..., bit_4 and c_p (where c_p acts as a bias, to judge whether the agent forgets or not). (2) Consequently, md_2 activates negatively, and (3) the plasticity of the learning of some connections which connect to std 0 (the declining neuron) is modulated by the positive signal from md_2. (4) Accordingly, the connections that connect to std_0 change positively, and (5) std_0 activates positively and the network declines the test.

**Figure 11:**
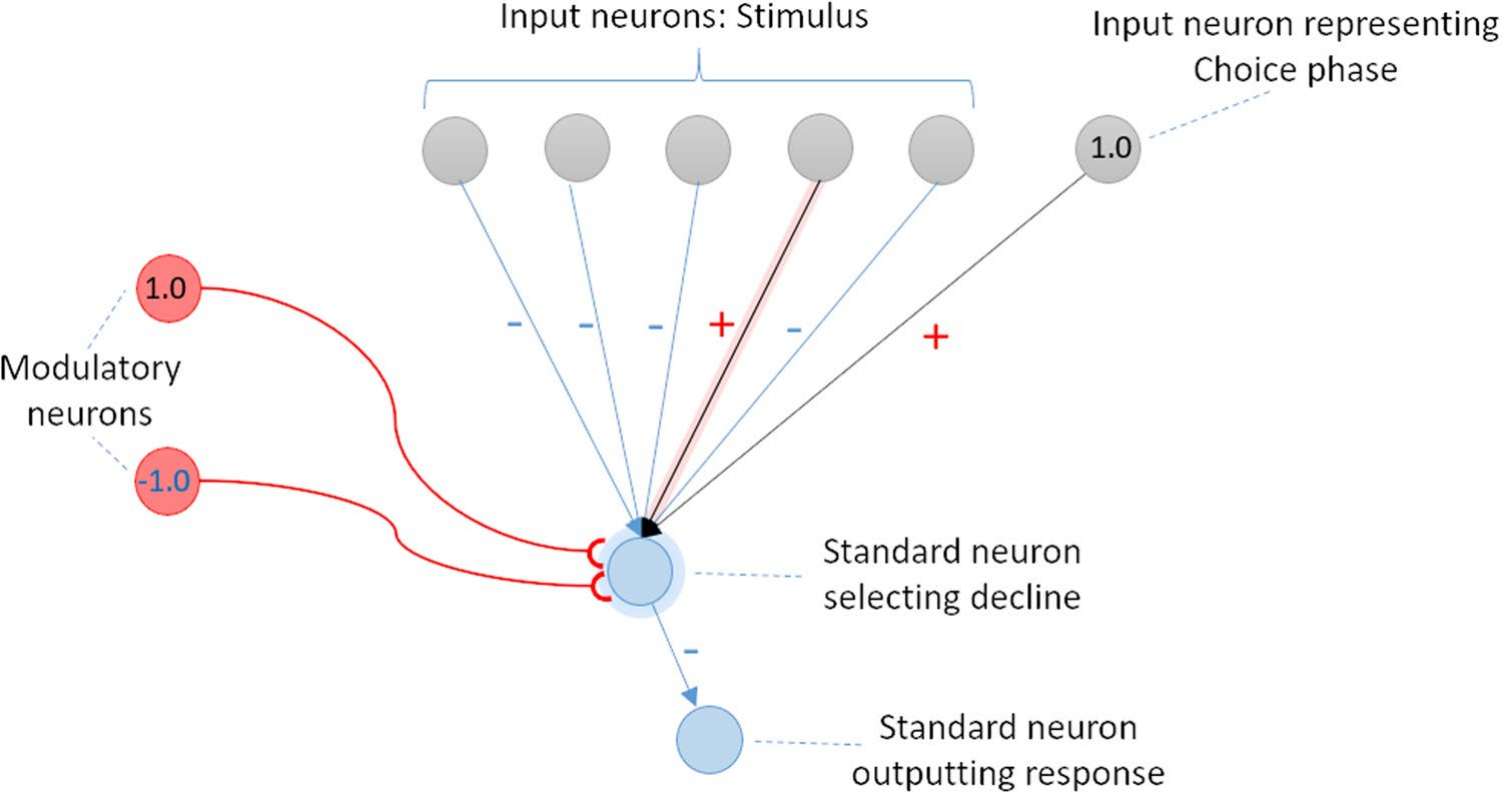
Main structure of an alternative first-order plasticity network capable of behaving like the evolved network. The network makes a metamemory judgment by directly using the result of a judgment whether the memory state exists or not without forming a representation.

**Figure 12:**
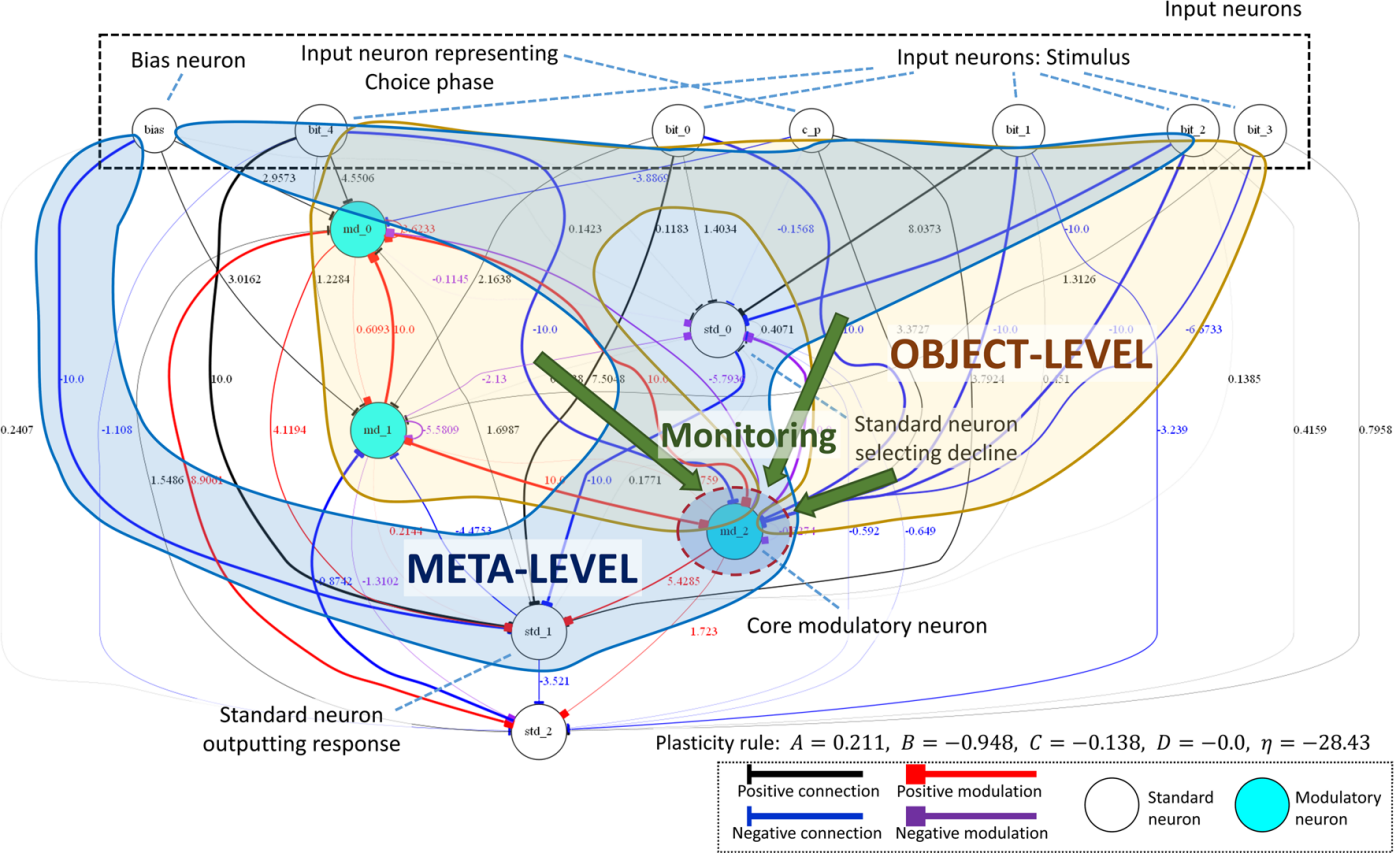
Internal structure of the evolved neural network and its correspondence to the structure of Nelson and Narens’s formulation.

How is this problem considered in human metacognition in general? Koriat^12^ summarized that humans cannot directly evaluate internal memory strength, and instead, they make certainty judgments by drawing inferences from many cues that they can evaluate (e.g., familiarity and ease of processing).

**Table 1:** Accuracy for each input pattern in each case ($delay = 40$).

Input patterns	Accuracy in forced tests	Accuracy in chosen tests (probability of taking)	Accuracy in declined tests (probability of declining)
00001	0.361	0.835 (0.270)	0.175 (0.730)
00010	0.354	0.803 (0.266)	0.216 (0.734)
00100	0.340	0.764 (0.240)	0.205 (0.760)
01000	0.361	0.819 (0.258)	0.183 (0.742)
10000	0.347	0.816 (0.272)	0.206 (0.728)

According to these arguments, there is no doubt that the results of Hampton’s DMTS experiments alone are insufficient in providing a substantial contribution to the discussion of metamemory. This is owing to the fact that there is a complex gap between the behavior or function of metamemory and the mechanisms that govern them. This is where the constructive approach comes into play, such that we have immediate access to all the phenomena occurring in the artificial neural networks on a computer, unlike the cases with animal or human subject experiments, although the questions of whether and what we can actually understand from the data are a different issue. This advantage is provided by the property that all computations are completed on a computer, and all computed information is fully accessible. We believe that this constructive approach can provide a new perspective to support the aforementioned arguments from relevant behavioral experiments. If we succeed in evolving a type of metamemory in silico, this does not only demonstrate a possible mechanism that satisfies the metamemory, but also demonstrates the *evolutionary plausibility* of the metamemory mechanisms that have been explored and interpreted in previous behavioral experiments. This could be an important contribution to demonstrate a certain validity for the metamemory mechanisms that have been discussed by previous researchers from the standpoint of evolutionary plausibility.

Based on the study by Sudo et al.^6^, Yamato et al. made a similar evolutionary model, and successfully demonstrated that there were two types of mechanisms in the evolved agents, either of which made them determined the metamemory ability from the perspective of the DMTS paradigm^13^. However, if we consider the aforementioned arguments in animal experiments, it would be difficult to claim that one of the types of evolved mechanisms has exhibited a metamemory ability. Specifically, this type exhibited a strategy that tends to decline the test when the input pattern was difficult to remember or recognize, independent of the state of its neural network, exactly as Call pointed out^9^. This strategy is purely public in Hampton’s classification. However, the other type was implemented by detecting the deterioration of the memory content (forgetting), but was not achieved by self-referencing its memory state. Instead, it was implemented by some kind of spurious relationship between its memory state and selection circuit of decline. More precisely, the metamemory judgment in this type of mechanisms is based on whether there is a change in its network, which implies that a substantial disturbance of cognitive activity has occurred in the network. This is a first-order explanation of an internal physiological state. This mechanism might roughly correspond to the memory flag hypothesis^14^ that a subject adopts an indicator for the presence of memory as a metacognitive response while not being aware of the memory content. This feature corresponds to one of the suggested features of human metamemory. Although the judgment based on this mechanism can be regarded as metamemory from this perspective, this mechanism does not meet the criterion of metamemory in the narrow sense^10^.

The contribution of our research is to introduce a new dimension of evolutionary plausibility based on a constructive approach to the previous discussions on non-human animal experiments, which typically consist of presenting each other with mechanisms that can explain the experimental results as sufficient conditions. When presented with a mechanism that can explain the results of an animal experiment, this dimension can create the perspective on determining the feasibility of an evolutionary scenario in which a mechanism implemented as an artificial neural network emerges, as well as determining the existence of an evolutionary scenario in which an alternative mechanism emerges. In addition, we believe that in the process of investigating this perspective, we can contribute to the development of a novel experimental paradigm.

Accordingly, we make two changes to the method for the evolution of neural networks^15^. Specifically, we add Gaussian noise to the input values of all neurons in the neural networks, such that robustness is required for the abilities of agents. We also provide a constant value to each of the pattern input neurons during the choice phase to explicitly indicate that the agent is in that phase. This study successfully demonstrates that by conducting a detailed analysis of the evolved neural networks, the evolved neural network satisfies the metamemory criterion in the narrow sense, presented, for example, by Carruthers^10^, and finally situates the results in Nelson and Narens’s conceptual formulation of metamemory^1,2^.

## Model

### Task

Figure 2 presents an overview of the task based on the DMTS paradigm used in^6^. First, an agent receives a target pattern comprising five binary digits, which is randomly selected from 00001, 00010, 00100, 01000, and 10000 in the study phase. The delay phase follows, in which the agent receives 00000 as a distractor pattern a predefined number of times defined by Eq. (1), which will affect the degree of uncertainty in its memory.

1 $$\begin{aligned} N_{delay} =\ \lfloor { \frac{-1}{\lambda \times \ln {(R)}} } \rfloor +\ 1, \end{aligned}$$

where $\lambda$ denotes a parameter related to the shape of the distribution, and *R* represents a uniform random number from [0, 1].

Subsequently, with a probability of 2/3, the choice phase commences. During this phase, the agent receives a signal, implying that it is in that phase, and a constant value (*CV*) which is given to each input neuron as an input pattern. The constant value is expected to be used for self-reference of a memory. One output from the agent in the range $[-1, 1]$ will be interpreted as an intention to decline or take the trial when it is more than 1/3. In this case, the agent receives a small reward (0.3). Otherwise, if the value equals or exceeds 1/3, it will be interpreted as an intention to take the test. However, with a probability of 1/3, the choice phase is skipped as a compulsory condition.

In the test phase, the agent receives all patterns successively in random order, and an output ranged in $[-1, 1]$ is interpreted as a response to each pattern. Specifically, when the response exceeds 1/3 for the first time, the corresponding input pattern will be interpreted as the selection of the agent and the task ends. If it matches the target pattern presented in the study phase, the agent is rewarded with a large reward (1.0). Otherwise, if it does not match the target or all responses do not exceed 1/3, it is rewarded with nothing (0.0).

In addition, we adopt an unsolvable condition, in which agents do not receive one of the five patterns but receive a distractor pattern 00000 in the study phase. In this condition, they receive the large reward (1.0) only if it selects the decline option. We expect this addition to accelerate the evolution of the ability to select the option.

### Metamemory criteria

Typically, the condition for an agent to have metamemory has been that it satisfies one of the behavioristic paradigms for metamemory (e.g., DMTS paradigm) without depending on external cues. Here, the term “external” (cues/information) refers to stimuli that are provided to the neural network as input values while “internal” refers to stimuli that do not directly use the input values, but are generated internally via the activity of the network itself in the model. However, there are multiple mechanisms that meet this condition as we discussed in the previous section. To distinguish them, we define the following logical criteria in terms of mechanisms based on the metamemory-based behavior selection^13^, as illustrated in Fig. 3. By applying these criteria, we can classify the mechanisms revealed by evolutionary experiments in computers and discuss their evolutionary plausibility.

- Criterion 1 (C1)
  *The agent satisfies one of the behavioristic paradigms for metamemory, but not by primarily altering the behavior according mainly to particular stimuli configurations.*

This criterion excludes some solutions in the experiments based on the DMTS paradigm, that have been criticized, and simultaneously, it does not require the agent to monitor stored information regarding the stimuli input.

- Criterion 2 (C2)
  *C1 is satisfied, but is based on the self-reference on some part of the stored information regarding the stimuli input.*

The agent is required to access even a portion of its memory although it is not enough to distinguish whether the achieved metamemory function is based on second-order representations or not.

- Criterion 3 (C3)
  *C2 is satisfied, but is based on judgments based primarily on internally constructed second-order representations.*

This criterion was set as the narrowest definition of metamemory. To meet this criterion, the agent should store information generated based on the state of memory representation (first-order representation) as meta-representation (second-order representation). This paper does not go into the details of human metamemory. However, as mentioned in the Introduction section, it has become clear that humans seem to make certainty judgments by drawing inferences from many cues that they can evaluate. Therefore, what is certain is that the human case satisfies the criteria up to C2.

### Neural network

An artificial neural network is a mathematical model inspired by the activity of nerve cells in a brain. Figure 4 illustrates the activation process of a standard neuron in a network, which determines the basic dynamics of the network. An entire network comprises neurons and directed connections with weight values between neurons. Each neuron (1) receives a weighted sum of incoming values from other neurons, and (2) outputs an activation value by applying a nonlinear transformation (hyperbolic tangent function) to the sum. This process is simultaneously conducted for all the neurons at each time step to calculate their current output values using the outputs from other neurons at the previous time step. We allow neurons to have recurrent connections that enable their outputs to affect their future inputs.

The neural network of each agent comprises several standard neurons including seven input and two output neurons, and modulatory neurons (described in the next subsection), as illustrated in Fig. 5. Among the seven inputs, one input neuron receives a signal indicating whether it is in the choice phase (1) or not (0). Another is a bias neuron that receives a constant input value (1.0). Each input of the other five neurons gets one of the five digits of an input pattern, respectively.

The topology of the network evolves while ensuring that the number of neurons is not more than 16, including standard and modulatory neurons, but excluding input neurons. Each connection weight of the network is allowed to have a range of $[-10, 10]$. The network inputs the same patterns sequentially for four times in the choice phase and three times in the other phases. The repetition of input is essential when recurrent networks are used to propagate the effects of inputs throughout the network. Each value of all neurons in all phases is slightly modified by adding a suitable amount of Gaussian noise (input neurons: $\mu = 0.0$ and $\sigma = 0.1$, other neurons: $\mu = 0.0$ and $\sigma = 0.0001$). The Gaussian noise is added to prevent the evolution of agents that are not robust. We consider the lack of robustness as the reason why the evolved agents met C1 but not C2^15^.

### Neuromodulation

We employ neural networks with neuromodulated plasticity^7^ to evolve metamemory ability, because we believe that neuromodulation is important in achieving higher-order cognitive functions. Specifically, Arnold et al.^16^ suggested the selection for learning and selection for second-order learning, i.e., change in (first-order) learning, as the causal factors driving the emergence of innate and acquired forms of representation, respectively, and successfully demonstrated that the cognitive map, which is one of the mental representations, can evolve via second-order learning based on second-order modulation. The neural network in our model also contains modulatory neurons, in addition to standard neurons. Modulatory neurons affect the learning rate of the connection weights of target neurons and dynamically change it, as illustrated in Fig. 6. In particular, the output of the modulatory neuron $m_i$ modulates the learning rate of the update rule for the connection weight by adopting it as a modulatory signal, instead of directly influencing an activation signal $a_i$. They are computed using Eqs. (2) and (3).

2 $$\begin{aligned}&a_i =\ \sum _{j \in Std} {w_{ji} \cdot o_j}, \end{aligned}$$

3 $$\begin{aligned}&m_i =\ \sum _{j \in Mod} {w_{ji} \cdot o_j}, \end{aligned}$$

where $w_{ji}$ is a connection weight from a presynaptic neuron *j* to a postsynaptic neuron *i*. *Std* and *Mod* represent the sets of standard and modulatory neurons connected to neuron *i*, respectively. $o_j$ denotes an output of the neuron *j* and is computed as $o_j =\ \tanh {(a_j)}$. We consider that the neuron is positively activated if its output value is positive (or approximately 1) and negatively activated if it is negative (or approximately $-1$). The connection weight from a neuron *j* to a neuron *i* is updated only when it is modulated by one or more modulatory neurons using Eq. (4), which is based on an extension of Hebb’s rule called the extended Hebbian rule^17^.

4 $$\begin{aligned} \Delta w_{ji} =\tanh {(m_i)} \cdot \eta \cdot (Ao_jo_i +\ Bo_j +\ Co_i +\ D), \end{aligned}$$

where $o_j$ and $o_i$ represent the outputs of a presynaptic neuron j and a postsynaptic neuron *i*, respectively. $\eta$, *A*, *B*, *C* and *D* are also genetic parameters. Hence, the update rule can represent various types of synaptic updating via the evolution of these parameters.

Here, we present an example of learning in a minimal neural network in Fig. 7. First, a neuron receives modulation signals as (1). Second, the network calculates weight magnitudes to be added to each connection weight based on the extended Hebbian rule as (2). Finally, the connection weights are updated as (3).

### Evolutionary algorithm

We employ a variant of a genetic algorithm. The setup of the algorithm is basically the same as that proposed in^6^. Each agent has a matrix of real-valued connection weights (one axis corresponds to presynaptic neurons while the other to postsynaptic neurons) and the type of each neuron (standard or modulatory) for the decision of the structure, and five parameters ($\eta , A, B, C, D$) for the update rule Eq. (4), as a genome. Each connection weight $w_{ij}$ and $\eta$ are in the range $[-100, 100]$ while $A, \ldots , D$ in $[-1, 1]$. These parameters in genotype are converted to each weight $w_{ji}$ in the phenotype using Eq. (5) and $A, \ldots , D$ in the phenotype using Eq. (6), except for $\eta$.

5 $$\begin{aligned}&w_{ji} =\ {\left\{ \begin{array}{ll} 0 &{} (|w_{ji}^3| <\ 0.1) \\ 10 \cdot w_{ji}^3 &{} (otherwise), \end{array}\right. } \end{aligned}$$

6 $$\begin{aligned}&p =\ {\left\{ \begin{array}{ll} 0 &{} (|p^3| <\ 0.1) \\ p^3 &{} (otherwise), \end{array}\right. } \end{aligned}$$

where $p \in \{A, B, C, D\}$. The total score of each agent obtained by performing the task of each is defined as its fitness. The genetic operators are conducted as follows. Individuals are stored in an array, and are divided into consecutive segments of size 5 (with a random segmentation offset at each generation). The best individual of each segment becomes a parent, and then generates 5 children for its segment by repeating the crossover with a probability of 0.1 or copying itself otherwise. When crossover happens, a partner individual is randomly selected from all individuals in the population. Random integers *r* and *c* are selected from [1, *N*], while the two matrices are generated by exchanging the sub-matrices of the parents that are composed of *i*, *j* elements with *i* and *j* less than or equal to *r* and *c*, respectively. A uniform crossover is performed on the parameters for the plasticity rule. Then, as a mutation operator, with a probability of 0.1, Gaussian noise ($\mu = 0.0$, $\sigma = 0.3$) is added to each of the connection weights and the parameters for the plasticity rule, except $\eta$, while Gaussian noise ($\mu = 0.0$, $\sigma = 3.0$) is added to $\eta$. Finally, the insertion, deletion and duplication of each neuron are independently performed with probabilities of 0.04, 0.06, and 0.02, respectively. When insertion happens, the weight of the added neuron is randomly set in the range $[-1, 1]$, and the neuron type (standard or regulatory) is randomly set. These processes constitute a generation and are repeated *G* times.

## Experiment and analysis

### Basic results and criterion C1

We performed ten evolutionary trials using the parameters: $N = 300$, $G = 1000$, $\lambda = 0.7$, and $CV = 0.25$. During the first 100 generations, the agents had to jump to the test phase by skipping the choice phase every time. This setting was meant to guide the evolution in the initial stage. In subsequent generations, the tasks with the unsolvable condition were randomly performed $U = 50$ times among $T = 300$ times of task execution. The neural network of each agent was initialized by setting random values within corresponding possible ranges whenever a task started.

We successfully identified neural networks that satisfy C3 in one trial among ten trials via the following analysis. Figure 8 illustrates the behavior of the evolved agents for each delay time in the delay phase. This individual exhibits a higher accuracy for the selection condition than that in the forcing condition. We can also observe the increase in the avoidance rate with the increase in the delay. This result can be explained by assuming that the agent has a metamemory function, and is also consistent with the behavior of the monkey in Hampton’s experiment^5^.

Table 1 presents the average values of accuracy with a time delay of 40 in forced, chosen, and declined tests (assuming that they had taken the test despite declining). The latter two are shown with the probabilities of taking and declining in parentheses, respectively. There is a certain difference in the accuracy between forced and chosen tests, and between chosen and declined tests, independent of input patterns. This indicates that the agent tended to avoid the trials, which it could not answer correctly. The behavior of this agent satisfies C1 because the behavior was not simply based on the response for particular input stimuli configurations.

### Criterion C2

We analyzed how the network had solved the metamemory task by observing temporal changes in neuronal activity and connection weights of the network. Figure 9 presents the network that was analyzed above. We determined that the network has a second-order modulator structure in which two modulatory neurons md_0 and md_1 (in light blue) modulated the plasticity of the connections from the standard neurons to another modulatory neuron md_2, as depicted in the yellow area in Fig. 9. This structure played an important role in memorizing a received pattern, declining the test, and answering the test as follows.

#### Memorizing and its protection

Figure 10a presents the mechanism for memorizing a received pattern. The network reflects and stores a target pattern, which it received in the study phase by assigning the value (0 or 1) of each bit of the pattern, as the sign of the corresponding connection weight. This ability is achieved by md_0 and md_1 maximally strengthening/weakening the connection weights between the five input neurons (from bit_0 to bit_4) and md_2 (hereinafter referred to as *core modulatory neuron*) by modulating the plasticity of these connections. During the delay phase, the two modulatory signals cancel each other out and the sum of the values approaches zero, which prevents the connection weights from changing. Hence, the memorized pattern tends to be protected. However, this mechanism breaks down when a large noise increases the absolute value of the sum of both values, again increasing the plasticity of the connections. In other words, the memorized pattern will be gradually forgotten after that. Notably, the stored weights basically tend to decrease towards the minimum value ($-10$).

#### Declining the test

Figure 10b presents the mechanism for declining the test. The core modulatory neuron manages the behaviors of declining and answering, depending on its activity state. It ascertains the presence of the memory for the presented pattern using the sign of the sum of the inputs coming from the six input neurons, five of them for input patterns and the rest for representing the Choice phase. Core modulatory neuron ascertains that the memory exists when the sign of the sum of inputs is plus; otherwise, it ascertains that memory does not exist. In the latter case, the core modulatory neuron changes the plasticity of related connections. Triggered by this, the learning makes the declining neuron activate positively. The network thus successfully declines the test when the memory content becomes faulty. In addition, we determined that the mechanism of pattern selection in the test phase is also based on the monitoring of memory state by the core modulatory neuron. In summary, the results of our experiments indicate that these evolved networks achieve a metamemory function based on the self-reference to the memory corresponding to the input pattern, in other words, they satisfy C2.

### Criterion C3

The memory part where the input pattern is stored and the part which modulates some connection weights by referring to the memory state is clearly separated in the network. The flow of information in this structure is consistent with Carruthers and Crystal’s criterion of metamemory as follows. In the Choice phase, the memory part first receives a constant value and outputs a certain value. This output value can be interpreted to represent the state (presence) of the memory. Second, the core modulatory neuron activates positively or negatively, depending on the value from the memory part, and alters the plasticity of some connections based on the activation, meaning whether the memory is maintained or not, respectively. Where the state of the connections formed by changing plasticity of connections using the core modulatory neuron is considered the representation of whether the network remembers the memory state or not. Such representation corresponds to knowledge about cognition of memory, i.e., metamemory in the narrowest sense (C3).

The fact that this structure comprises second-order plasticity via second-order modulation implies that second-order plasticity plays an important role in the realization of metamemory ability in a narrow sense. If a network has only first-order plasticity, it would exhibit a behavior based on first-order explanations. For example, let us consider an alternative network in which a declining neuron connects negatively to answering neurons, as illustrated in Fig. 11. At first glance, like the evolved network we analyzed, it has declining and answering abilities based on the results of memory state referencing. However, they are not based on the representation of the judgment (second-order representation) based on the memory state reference. The metamemory achieved by the network does not satisfy C3 while satisfying C2.

### Discussion

Finally, we point out the approximate correspondence between the above structure and the metacognition structure of Nelson and Narens’s model (Fig. 1) that is often used to integrally elucidate the processes of multiple aspects of metacognition integrally. Their model explains that the monitoring mechanism is achieved as follows: the meta-level monitors the state of the object-level, updates the internal model including the knowledge about its own cognition based on the result of monitoring, and determines the meta-level policy based on the state of the internal model^1,2^. According to the secondary representational structure and its flow of information, the evolved mechanism of metamemory can be reinterpreted as follows (Fig. 12). The part considered to store the input pattern corresponds to the object-level, the part observing the state of the memory part, including the core modulatory neuron and some connections of which weights are modulated by the core modulatory neuron, corresponds to the meta-level, and the state of the core modulatory neuron (meaning the presence of the memory content) corresponds to a model of the object-level inside the meta-level. This approximate structural correspondence might imply that networks with a similar structure can evolve to pass not only DMTS-based tasks that correspond to feeling-of-knowing (FOK) metamemory judgment but also other tasks that correspond to other metamemory judgments^1,2^, including judgment-of-learning (JOL) and confidence judgment (CJ), because Nelson and Narens’s model can be used to explain many aspects of metacognition.

## Conclusion

Our objective is to make a substantial contribution to the discussion on the mechanisms of metamemory. Accordingly, in this study, we conducted evolutionary experiments using the constructive methodology of previous studies^13,15^, comprehensively analyzed the metamemory mechanism of the evolved neural network in detail, and clarified the significance of the evolved metamemory. When discussing its significance, we used the criteria (C1, C2, and C3) for metamemory, which can exclude the solutions that had been criticized in the discussion on the experiments, based on the DMTS paradigm that had been often used to identify metacognition in non-human animals.

We successfully identified an agent that satisfies C3 and investigated the evolved neural network of the agent by analyzing its behavior, structure, and neural dynamics. First, we found that the agent exhibits behavior similar to a monkey that was claimed to have an ability of metamemory in Hampton’s experiment, and that the behavior is not a reflex behavior for particular input stimuli configurations. Then, we observed temporal changes in the state of the network, and demonstrated that the network has a structure in which two modulatory neurons modulate a few connections from the standard neurons to another modulatory neuron. We demonstrated that this structure plays an important role in memory and metamemory abilities as follows. In the study phase, the network reflects and maintains the structure of the received pattern in each connection weight between the corresponding input neuron and the modulatory neuron by the coupling effect of two modulatory neurons. Moreover, in the Choice phase, the core modulatory neuron modulates the plasticity of some part of connections that connect to the standard neurons for the output based on a monitoring result of the memory state. This modulation allows the network to appropriately decline or answer the test. A series of these processes realize metamemory based on self-reference. Finally, we presented a good correspondence between the evolved network structure and Nelson and Narens’s formulation, which is regarded as an essential feature of the networks that satisfy C3.

Our study introduced a new dimension of evolutionary plausibility to the previous discussion on non-human animal experiments. The obtained experimental results, which present a scenario of the metamemory evolution, based primarily on internally constructed second-order representations, can be interpreted to support the relationship between solving the DMTS task and the existence of metamemory utilizing internal cues in animal experiments. We believe that our study can contribute to the understanding of human metamemory and, furthermore, to the realization of artificial consciousness.

## Data Availability

The source code and the data used in this study are available at https://github.com/yamatakeru/Evolurionary_experiment_with_DMTS.
